# Antitumor properties of traditional lactic acid bacteria: Short-chain fatty acid production and interleukin 12 induction

**DOI:** 10.1016/j.heliyon.2024.e36183

**Published:** 2024-08-13

**Authors:** Parinaz Mobasherpour, Masoud Yavarmanesh, Mohammad Reza Edalatian Dovom

**Affiliations:** Department of Food Science and Technology, Faculty of Agriculture, Ferdowsi University of Mashhad, Mashhad, Iran

**Keywords:** Lactic acid bacteria, Probiotic, Antitumor compounds, Short-chain fatty acids, IL 12

## Abstract

This paper presents an *in vitro* evaluation of antitumor properties through producing short-chain fatty acids and inducing interleukin 12. In addition, it offers the most important and functional probiotic properties of 24 *Lactobacillus gasseri*, *Lactiplantibacillus plantarum, Lactobacillus acidophilus,* and *Limosilactobacillus fermentum* strains isolated from humans, foods, and fermented foods. To this end, survival in an acidic environment (pH = 2.5), tolerance in bile salt, viability in the presence of pepsin-pancreatin, adhesion percentage, antibiotic resistance, auto-aggregation, and potential percentage of co-aggregation are studied in contact with three human intestinal pathogens. These pathogens are *Escherichia coli* O157: H7 NCTC 12900, *Salmonella enterica* subsp*. enterica* ATCC 13076, and *Listeria monocytogenes* ATTC 7644. Also, *in vitro* induction amount of IL-12 in mouse splenocytes is investigated to evaluate antitumor properties by 19 strains of *L. gasseri* and *L. plantarum* along with the development of short-chain fatty acids (SCFA) by 5 strains of *L. fermentum* and *L. acidophilus*. Gas Chromatography Flame Ionization Detector (GC-FID) and enzyme-linked immunosorbent assay (ELISA) were used to measure short-chain fatty acids and IL-12, respectively. All strains had high viability under acidic conditions. The highest levels of pancreatin and pepsin resistance were found in strains LF56, LF57, LF55, OF, and F and strains LF56, LF57, and A7, respectively. All strains except LF56 had high resistance to bile salts. *L. gasseri* 54C had the highest average adhesion score (hydrophobicity) of 62.9 % among 19 strains*.* Despite the susceptibility of different strains of *L. plantarum* to the tested antibiotics, M8 and M11, S2G, A7, LF55, LF57, and 5G were resistant to kanamycin and chloramphenicol, respectively. Also, 21G was resistant to ampicillin, LF56 to tetracycline and M8, and M11, LF56, and 21G to Erythromycin. In addition, *L. gasseri* showed moderate resistance to ampicillin, erythromycin, and tetracycline, while *L. fermentum* ATCC 9338 showed good resistance to ampicillin, erythromycin, and chloramphenicol. In this respect, *L. plantarum* LF56 and *gasseri* 54C had the highest average auto-aggregation and co-aggregation against three pathogenic bacteria, respectively. The highest and lowest levels of acetic acid as short-chain fatty acids were produced by *L. fermentum* 19SH isolated from Horre 41.62 and *L. fermentum* 21SH from fermented seeds 27.047, respectively*.* Moreover, *L. fermentum,* with the OF code of traditional-fermented food origin, produced the most isobutyric acid, butyric acid, and valeric acid, with values of 0.6828, 0.74165, and 0.49915 mmol, respectively. *L. fermentum* isolated from the human origin with code F produced the most isovaleric acid of 1.1874 mmol. All the tested strains produced good propionic acid except *L. fermentum* 21SH from fermented seeds. Among strains, *L. plantarum* M11 isolated from milk and *L. gasseri* 52B from humans had the highest *in vitro* induction of IL-12, which is probably related to their cell wall compositions and structure.

## Highlights

• *L. gasseri* 54C and *L. plantarum* LF55 and LF56 were the strongest probiotic strains.
• *L. fermentum* 19SH and OF produced the highest amounts of short-chain fatty acids.
• *L. plantarum* 8SH and M11 and *L. gasseri* 52B showed the maximum induction of IL-12.
• Having a thicker bacterial cell wall causes more SCFA and IL-12 to produce.

## Graphical abstract

**Figure:**
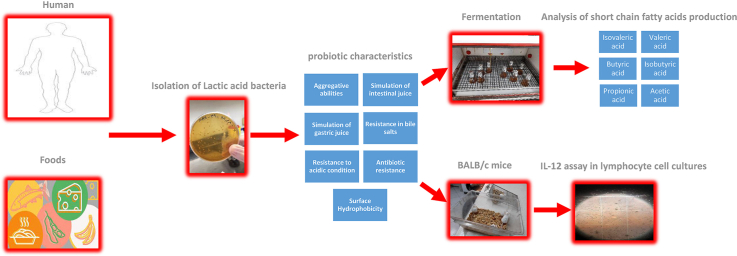

## 1 Introduction

Probiotics are products containing an equivalent amount of living and advanced microorganisms that colonize and modify the microbiota of the host body, resulting in a beneficial effect [1]. Regarding the positive effect of probiotics on the host body, they must be able to survive for a long time in the gastrointestinal tract. Probiotics are used to activate the immune system and inhibit certain infections, in addition to their use as growth stimulants [2]. These compounds prevent cancer by influencing animal and human digestive enzymes, suppressing tumors and metabolites *in vitro*, and inhibiting carcinogens *in vivo* and *in vitro* [3]. Probiotic bacteria have different functions depending on their origin.

*In vitro*, animal, human, and epidemiological experiments have shown that certain *Lactobacillus* strains can minimize the risk of different cancers by developing fermented products and preventing tumor development [4]. Lactobacilli extracted from fermented milk products can enhance lactose digestion, regulate serum cholesterol levels, and control gastrointestinal diseases, certain forms of cancer in various medical and health areas (e.g., managing pregnancy infections), antibiotic-related diarrhea, and intestinal inflammation. In addition, they are effective in treating allergic diseases, preventing urinary tract infections, stimulating the immune system, and stabilizing the intestinal microbiota [5]. Lactic acid bacteria isolated from yogurt also increase the immune system, reduce gastrointestinal infections, and lower cholesterol, cancer, and diarrhea.

Short-chain fatty acids are among the antitumor compounds developed by probiotic bacteria. These acids are classified as volatile fatty acids with 1–6 carbons that can be straight or branched and are absorbed in the large intestine. Acetic acid is the most concentrated short-chain fatty acid in the large intestine [6]. Kahouli et al. [7] studied *L. fermentum* NCIMB 5221 for its ability to generate fatty acids and antitumor activity. These authors reported the antitumor production of the probiotic bacterium *L. fermentum* NCIMB 5221 as a producer of ferulic acid (FA) and an antioxidant and antitumor compound with the capacity to generate free fatty acids (FFAs). Wang et al. [8] investigated the synthesis of short-chain fatty acids by lactobacilli using *Lactobacillus acidophilus* RD758. According to this study, the fermentation temperature influences the fatty acid content of the *L. acidophilus* RD758 membrane, and the fermentation pH affects the concentration and form of fatty acid.

White blood cells and other cells secrete cytokines, as some low molecular weight proteins or glycoproteins, in response to certain stimulants. Interferon-gamma (IFN-γ) has shown an anti-proliferative and apoptotic induction effect on many receptor-induced tumor cells, indicating its defensive function against many tumors. Lactic acid bacteria, especially colon probiotic bacteria, promote health and modulate host immune responses [9]. Tamang et al. [10] pointed out that synthesizing anti-inflammatory cytokines (e.g., IL-12 and IFN-γ) modulates T helper type 1/T helper type 2 (Th1/Th2) immune cells.

*L. gasseri* OLL2809 is a lactic acid bacterium that actively induces IL-12 (p70) [11]. Also, the helper T cell is a type of T lymphocyte and white blood cell with a significant role in the body's immune system. Since IL-12 (p70) promotes the division of simple cells into Th1 cells, it can be used to assess *Lactobacillus* probiotics' anti-inflammatory effect [11]. Research has shown that *L. plantarum* L137 *and L. plantarum* JCM1149 are active IL-12 (p70) inducers that can trigger Th1-type immune responses with anti-inflammatory and antitumor effects [12].

The present study aims to screen four strains of traditional lactic acid bacteria (*L. gasseri*, *L. plantarum, L. fermentum*, and *L. acidophilus*) with antitumor properties in terms of producing short-chain fatty acids and inducing interleukin 12 and the relationship of these characteristics with probiotic properties.

## 2 Materials and methods

### 2.1 Bacterial strains, media, and growth conditions

Bacterial strains used in this study (i.e., human and conventional foods samples) were described using molecular methods according to the 16srRNA gene sequence in Table 1 (microbial bank, Department of Food Science and Technology, Faculty of Agriculture, Ferdowsi University of Mashhad). Also, indicator microbial pathogens to study some probiotic properties were *E. coli* O157: H7 NCTC 12900, *S. enterica* subsp*. enterica* ATCC 13076, and *L. monocytogenes* ATCC 7644. Lactic acid bacterial strains were cultured overnight at 37 °C in De Man Rogosa Sharpe (MRS) broth and agar)Ibresco, Iran). Microbial pathogens were cultured overnight at 37 °C in Mueller-Hinton agar and Brain Heart Infusion broth (BHI))Ibresco, Iran) culture media. Among lactic acid bacteria, *L. acidophilus* AC-ATCC 4356 and *L. fermentum* (OF, F-ATCC 9338, 19SH, and 21SH) were used to produce short-chain fatty acids. Meanwhile, *L. gasseri* (52B, 49A, 47B, and 54C) and *L. plantarum* (LF48, LF55, LF56, LF57, M8, M11, S2G, A7, D1, 21G, 5G, 8SH, 10SH, 11SH, and 61G) were used to induce IL-12.

**Table 1:** Characteristics of lactic acid bacteria strains used in this study.

Number	Species	Source	Strain no./ref./accession no.
1	*Lactiplantibacillus plantarum*	Lighvan cheese	LF48 [13]
2	*Lactiplantibacillus plantarum*	Lighvan cheese	LF55 [13]
3	*Lactiplantibacillus plantarum*	Lighvan cheese	LF56 [13]
4	*Lactiplantibacillus plantarum*	Lighvan cheese	LF57 [13]
5	*Lactiplantibacillus plantarum*	Milk	M8 KP212404
6	*Lactiplantibacillus plantarum*	Milk	M11 KP212405
7	*Lactiplantibacillus plantarum*	Wheat bran	S2G NR104573.1
8	*Lactiplantibacillus plantarum*	Small intestine of the infant	A7 KC355240
9	*Lactiplantibacillus plantarum*	Sourdough	D1 [14]
10	*Lactiplantibacillus plantarum*	Camel milk	5G KM495894.1
11	*Lactiplantibacillus plantarum*	Fermented olives	21G [15]
12	*Lactiplantibacillus plantarum*	Sauerkraut	8SH^a^ (ATCC14917)
13	*Lactiplantibacillus plantarum*	Tarkhine	10SH^a^
14	*Lactiplantibacillus plantarum*	Infant feces	11SH^a^
15	*Lactiplantibacillus plantarum*	Pitcher cheese	61G KM495875.1
16	*Lactobacillus gasseri*	Vaginal	52B KP090115
17	*Lactobacillus gasseri*	Vaginal	49A KP090114
18	*Lactobacillus gasseri*	Vaginal	47B KP090116
19	*Lactobacillus gasseri*	Vaginal	54C KP090117
20	*Limosilactobacillus fermentum*	Camel Doogh	OF [14]
21	*Limosilactobacillus fermentum*	Human	F (ATCC 9338)
22	*Limosilactobacillus fermentum*	Horre^b^	19SH^a^
23	*Limosilactobacillus fermentum*	Fermented seeds	21SH^a^ (ATCC 14931)
24	*Lactobacillus acidophilus*	Human	AC (ATCC 4356)

a The beneficial properties of probiotics have already been proven (Joghataei et al., 2019).

b It is a fermented food in southern Iran, including wheat and vegetable.

### 2.2 Investigation of probiotic characteristics

#### 2.2.1 Resistance of LAB strains to acidic conditions (low pH)

The strains were cultured for 48 h at 37 °C in MRS broth and centrifuged for 15 min at 6000×*g*. The supernatant was discarded, followed by washing the cells and suspending them in a phosphate buffer solution at pH = 2.5. After incubating at 37 °C for 4 h, the samples were diluted in sterile saline solution (0.85 % sodium chloride). Afterward, 100 μL of the last three dilutions were cultured on MRS agar, and the plates were incubated at 37 °C for 48 h. Finally, bacterial viability was measured using Eq. (1) [16]:

(1) $$survival\%=\frac{\log\;cfu\;of\;viable\;cells\;survived}{\log\;cfu\;of\;initial\;viable\;cells\;inoculated}\times 100$$

#### 2.2.2 Resistance of LAB strains in bile salts

The MRS broth culture medium containing 0.3 % (w/v) of bile salts (bovine bile (Ibresco, Iran)) with 100 μL of grown bacteria was incubated for 4 h at 37 °C. Before and after incubation, 100 μL of successive dilutions was cultured on the surface of MRS agar and incubated for 48 h at 37 °C. The bacterial survival percentage was determined using Eq. (1) [17].

#### 2.2.3 Simulation of gastric juice and LAB strains resistance

About 100 μL of grown bacteria was inoculated into the simulated gastric solution (3 mg/mL pepsin in 0.85 % sterile saline solution at pH = 2.5) and incubated at 37 °C for 4 h. Live cell count before and after incubation was determined by culturing 100 μL of successive dilutions on the surface of MRS agar medium and its incubation at 30 °C for 48 h [18].

#### 2.2.4 Simulation of intestinal juice (pancreatin) and LAB strains resistance

Intestinal juice was prepared with 1 mg/mL pancreatin in 0.85 % saline solution (pH = 8). For this purpose, 100 μL of exponential phase bacteria was inoculated into pancreatin solution and kept at 37 °C for 6 h in an incubator. Afterward, live cell count before and after incubation was determined by preparing serial dilutions and culturing 100 μL of dilutions on MRS agar medium superficially. Cultured mediums were incubated at 30 °C for 48 h [18].

#### 2.2.5 Surface hydrophobicity of LAB strains

The exponential phase of bacteria was assessed by incubating the strains for 18–24 h at 30 °C in an MRS broth culture medium and then centrifuged at 5000×*g* for 10 min. The supernatant was discarded, and the cells were washed twice with 50 mM K_2_HPO_4_ buffer at pH = 6.5. In the next step, the cells were suspended in phosphate-buffered saline (PBS; Pharmed, Iran) such that the cell density range was between 0.8 and 1 (spectrophotometer, lightwave S2000UV/Vis). About 3 mL of the microbial solution was mixed with 0.6 mL of n-Hexadecane into a test tube for 120 s, and the tubes were incubated at 37 °C for 30 min to separate into two phases. The upper phase was then cautiously separated, and the absorption of the lower phase was measured at a wavelength of 560 cm^−1^ [19].

(2) $$H\%=\frac{A0-A}{A0}\times 100$$

A_0_: Absorbance in the range of 0.8–1.

A: Lower phase absorption.

#### 2.2.6 Antibiotic resistance

This experiment was carried out in 96-well cell culture plates using the broth microdilution method. First, 95 μL of MRS broth was added to each well. Except for the positive control row, 100 μL of ampicillin, kanamycin, erythromycin, chloramphenicol, and tetracycline antibiotics with an initial concentration of 1 mg/L were poured into the first well of each row, and then from the first to the second well, and so on. Dilution was completed up to the twelfth well. Next, 5 μL of each tested strain was added to each well, except to the negative control row, and the cell culture plates were kept at 37 °C for 24 h. The turbidity of the positive and negative control wells before warming and the rest after warming were read by ELISA reader (Stat fax 2100, USA). The lowest inhibitory concentration in the first well, lower than the positive control, was considered MIC. It is of note that positive and negative control wells do not contain antibiotics and bacteria, respectively [20].

#### 2.2.7 Aggregative abilities of LAB strains

Auto-aggregation and co-aggregation of strains were investigated according to the method by Del et al. [21]. Under the microaerophilic conditions, the strains were grown for 24 h at 37 °C in an MRS broth culture medium. After centrifugation (sigma 3–30K, Germany(at 1372×*g* for 10 min, the pellets were redissolved in 10 mL of PBS to around 10^8^ CFU/mL (0.2 - 0.3OD at 550 nm WL). The suspensions were mixed for 10 s and left at room temperature for 6 h without shaking. The absorbance was measured using a spectrophotometer at 600 nm while 1 mL of the mentioned suspension was collected every hour. The following formula was used to quantify the auto-aggregation percentage [20].

(3) $$\text{Auto}-\text{aggregation}=1-\left(\frac{\text{At}}{A0}\right)\times 100$$

At: Absorption at different times.

A0: Absorption at time zero.

In this step, 3 mL of bacterial suspensions were subjected to experiments. Next, bacterial pathogens (i.e., *E. coli* O157: H7 NCTC 12900, *S. enterica* subsp*. enterica* ATCC 13076, and *L. monocytogenes* ATCC 7644) with a concentration of 10^8^ CFU/mL and absorption (0.25-0.05OD at 600 nm WL) were mixed for 10 s and left at room temperature for 6 h without shaking to evaluate co-aggregation. Samples containing 6 mL of a bacterium suspension were used as a control. After a 6-h incubation at room temperature, 1 mL of this suspension was collected every hour, and the absorptions of the mixed suspension (probiotics and pathogens) and control were measured separately. The co-aggregation percentage was determined using Eq. (4), where AX and AY are the aggregations of bacterial strains and pathogens experimented with, respectively. Also, A (X + Y) denotes the total aggregation of bacterial strains and pathogens together [21].

(4) $$\text{Co}-\text{aggregation}\%=\frac{\left(\left(\text{Ax}+\text{Ay}\right)/2\right)-A\left(x+y\right)}{\text{Ax}+\text{Ay}/2}\times 100$$

### 2.3 Preparation of bacteria for producing short-chain fatty acids

Bacterial strains (*L. fermentum* with codes OF, F-ATCC9338, 19SH, 21SH, *and L. acidophilus* with code AC-ATCC4356) were transported to a fermentation container with 100 mL MRS broth medium and placed in an incubator at 37 °C. Next, they were shaken in the incubator at 4×*g* for 24 h to produce short-chain fatty acids, followed by transferring the culture medium to 50 mL of falcons. Because of creating the same conditions for all strains to produce a short-chain fatty acid, the same concentration (number of bacteria/unit volume) with an absorbance of 0.8 at 660 nm equal to 3.6 × 10^6^ CFU/mL of bacterial strains was considered in the study. After 24 h of fermentation, the medium was centrifuged for 10 min at 2800×*g* at a constant temperature of 4 °C to separate short-chain fatty released into the culture. The supernatant containing fatty acids was transferred to a 2-mL microtube and placed in a freezer at −80 °C for 24 h [7].

### 2.4 Derivation of short-chain fatty acids

After thawing the frozen samples, they were centrifuged at 30 rpm for 30 min. The supernatant was then poured into a sterile microtube containing 300 μL of meta-phosphoric acid (Honeywell, Sweden) and vortexed. After that, the microtube was kept at 25 °C for 25 min and subsequently centrifuged at 2800g for 15 min. Next, the samples derived for analyzing short-chain fatty acids (SCFA) were injected into a GC-FID (Gas Chromatography-Flame Ionization Detector). The total contents of SCFA, propionate, acetate, and butyrate were calculated. Starting at 250 °C, the process was as follows: the sample (5 μl) was injected into the capillary column (Agilent HP-5 ms, the USA) at a split ratio of 25:1, which had been covered with a film, 0.15 μm in thickness, made from 80.2 % 1-methylnaphthalene. The nitrogen flow rate in the mobile phase was first set at 1 mL/min and maintained at this value for 1 min. After that, it shifted to 0.8 mL/min for 1 min, then to 0.6 mL/min for 1 min, and finally back to 1 mL/min for 9.2 min. The FID temperature was adjusted at 260 °C. Next, the synthetic air and helium flow rates were changed to 30 and 350 ml/min, respectively. Lastly, the oven temperature was set at 100 °C, kept for 7 min, subsequently increased to 200 °C at a rate of 25 °C/min and maintained for 5 min. The concentrations of the SCFA were expressed as μmol/ml [22].

### 2.5 Induction of IL-12 (p70) by *L. plantarum* and *L. gasseri* strains

Five-week-old male BALB/c mice weighing 24 ± 1 gr were purchased from Iran's Pasteur Institute and kept on a regular diet. In all experiments, mice between the ages of 6 and 9 weeks were used. It is of note that Iran's Pasteur Institute has approved the experimental protocols used. Also, the code of ethics IR.UM.REC. 1400.006 was received from the Biomedical Committee of the Ferdowsi University of Mashhad.

#### 2.5.1 Preparation of heat-killed bacteria

The bacterial strains were incubated in MRS broth for 24 h at 37 °C and centrifuged at 4200×*g* for 4 min at 4 °C. Next, the pellet was washed with saline solution and distilled water three times. After making a pellet suspension in distilled water, 50 μL of bacteria suspension was inoculated into 5 mL of MRS broth culture medium (1 % V/V) with a (pH = 6.4) and incubated for 18 h at 37 °C. Afterward, the culture medium was added to 100 mL of MRS broth (Erlenmeyer flask) and shaken at 4×*g* at 37 °C for 18 h in a shaker incubator. The pH of the culture medium was regularly checked and kept at 6.4 or higher. The contents of the Erlenmeyer flask were placed into a pre-weighed falcon and centrifuged at 1000×*g* at 4 °C for 15 min. After discarding the supernatant, the pellet was washed twice with saline solution and once with distilled water (pellet weight was measured each time). Finally, the suspended pellet (2 mL in deionized water) was heated at 75 °C for 60 min in a hot water bath (MEMMERT WNE45). The killed cells were lyophilized and maintained to assay for producing IL-12 (p70) *in vitro* [11].

#### 2.5.2 IL-12 assay in lymphocyte cell cultures *in vitro*

BALB/c mice (n = 5) were sacrificed by excising their spinal cords and aseptically removing their spleens. The spleens were then teased apart with tissue forceps in 10 mL of RPMI-1640 medium (Betacell BE25500) containing 10 % (v/v) heat-inactivated fetal bovine serum (FBS; Betacell BE31100) supplemented with 100 μg/mL penicillin and streptomycin (10 % FBS-RPMI 1640). They were then centrifuged for 5 min at 450×*g*. Erythrocytes were lysed in a buffer containing 0.826 gr NH4CL, 0.119 gr NaHCO3, and 20 μl EDTA 0.5 M (pH = 8) in 100 mL of solution with a pH = 7.2 to 7.3. After adding 10 mL of 10 % FBS-RPMI 1640, the cells were centrifuged (Eppendorf, Germany) at 450×*g* for 5 min and then counted. Lymphocyte cells (2.5 × 10^6^ cells/mL) were cultured in 24-well tissue culture plates in 10 % FBS-RPMI 1640 medium at 37 °C in the absence (control) or presence (1 g/mL lyophilized bacterial cells). After 2 days, the tissue culture supernatants were collected and analyzed using an ELISA kit (Mouse IL12/P70 Elisa kit. *Cat.No.E0020Mo*) to determine IL-12 (p70) production levels. Positive control contained 20 μL of PHA (Phytohaemagglutinin) to stimulate cytokine, and negative control was a well with no bacteria [11].

### 2.6 Statistical analysis

Data were expressed as the mean ± standard deviation. Each experiment was performed in triplicate. Statistical differences between or among the groups were analyzed using the one-way analysis of variance (ANOVA) with the Tukey mean comparison test. Differences were considered significant when the P-value was less than 0.05. The graphs were generated using GraphPad Prism software.

## 3 Results

### 3.1 Acid tolerance of LAB strains

All strains of *L. plantarum, acidophilus, fermentum*, and *gasseri* showed tolerance to acidic conditions at a pH of 2.5. *L. plantarum* isolated from milk (i.e., M8, LF56, and LF57) and *L. gasseri* isolated from a healthy female vaginal (i.e., 54C) had the highest resistance to acid among these strains (Table 2).

**Table 2:** Acid tolerance of LAB strains in PBS (PH = 2.5).

Species	Strains number	Source	Initial counts time (0h) cfu/ml	Log) cfu/ml)	Survival after time (4h) cfu/ml	Log) cfu/ml)	Survival%
***L. plantarum***	M8	Milk	6.6 × 10^7^	7.82 ± 0.01 ^bc^	7.9 × 10^7^	7.90 ± 0.00^a^	100.00
	M11	Milk	4.3 × 10^7^	7.63 ± 0.01^c^	1.6 × 10^7^	7.20 ± 0.01 ^cd^	94.38
	S2G	Wheat bran	2.1 × 10^8^	8.32 ± 0.00^a^	7 × 10^7^	7.85 ± 0.00^a^	94.27
	A7	Small intestine	0.86 × 10^7^	6.93 ± 0.00^d^	0.18 × 10^7^	6.26 ± 0.00 ^fg^	90.21
	LF56	Lighvan cheese	1.1 × 10^7^	7.04 ± 0.01^d^	1.2 × 10^7^	7.08 ± 0.02 ^cd^	100.00
	LF48	Lighvan cheese	1.4 × 10^8^	8.15 ± 0.02 ^ab^	1.1 × 10^6^	6.04 ± 0.01^gh^	74.16
	LF57	Lighvan cheese	1.5 × 10^7^	7.18 ± 0.00^d^	3.6 × 10^7^	7.56 ± 0.01^b^	100.00
	LF55	Lighvan cheese	1.1 × 10^7^	7.04 ± 0.01^d^	0.77 × 10^6^	5.88 ± 0.14 ^hi^	83.61
	D1	Sourdough	4.3 × 10^7^	7.63 ± 0.002^c^	2.5 × 10^6^	6.40 ± 0.01^f^	83.81
	21G	Fermented olives	1.4 × 10^8^	8.15 ± 0.001^ab^	1.1 × 10^6^	6.04 ± 0.11 ^gh^	74.16
	5G	Camel milk	1.6 × 10^8^	8.20 ± 0.02 ab	1.1 × 10^7^	7.04 ± 0.00 ^cd^	85.83
	61G	Pitcher cheese	1.3 × 10^8^	8.11 ± 0.01 ^ab^	0.54 × 10^6^	5.73 ± 0.1^i^	70.68
***L.gassseri***	54C	Vaginal	0.18 × 10^7^	6.26 ± 0.00^e^	0.96 × 10^7^	6.98 ± 0.00 ^de^	100.00
	49A	Vaginal	1.4 × 10^8^	8.15 ± 0.00 ^ab^	1.5 × 10^7^	7.18 ± 0.01 ^cd^	88.09
	47B	Vaginal	7.2 × 10^7^	7.86 ± 0.01 ^bc^	2.5 × 10^6^	6.40 ± 0.01^f^	81.43
	52B	Vaginal	8.1 × 10^7^	7.91 ± 0.00 ^bc^	1.9 × 10^7^	7.28 ± 0.02^c^	92.04
***L. fermentum***	OF	Camel Doogh	1.3 × 10^8^	8.11 ± 0.01 ^ab^	3.6 × 10^7^	7.56 ± 0.00^b^	93.13
	F	ATCC 9338	3.4 × 10^7^	7.53 ± 0.1^c^	0.81 × 10^6^	5.91 ± 0.00 ^hi^	78.46
***L. acidophilus***	AC	ATCC 4356	7.8 × 10^7^	7.89 ± 0.00 ^bc^	6.5 × 10^6^	6.81 ± 0.01^e^	86.33

Values are expressed in mean ± standard deviation.

Different letters in each column indicate significant differences (P < 0.05).

### 3.2 Bile salt tolerance of LAB strains

Except for L. *plantarum* isolated from Lighvan cheese (LF56), almost all strains had a 100 % survival rate in the simulated bile salt of the pancreas (Table 3).

**Table 3:** Bile salt tolerance of LAB strains.

Species	Strains number	Source	Initial counts time (0h) cfu/ml	Log) cfu/ml)	Survival after time (4h) cfu/ml	Log) cfu/ml)	Survival%
***L. plantarum***	M8	Milk	3.6 × 10^6^	6.56 ± 0.01 ^fg^	2.5 × 10^6^	6.40 ± 0.00^h^	97.58
	M11	Milk	2.2 × 10^6^	6.34 ± 0.00^g^	2.5 × 10^6^	6.40 ± 0.01^h^	100.00
	S2G	Wheat bran	1.3 × 10^7^	7.11 ± 0.00 ^cd^	4 × 10^7^	7.60 ± 0.01^c^	100.00
	A7	Small intestine	5.01 × 10^7^	7.70 ± 0.00^b^	7 × 10^6^	2.62 ± 0.00^j^	34.07
	LF56	Lighvan cheese	0.00	0.00^j^	0.00	0.00^k^	0.00
	LF48	Lighvan cheese	1.2 × 10^7^	7.08 ± 0.01 ^cd^	4.9 × 10^6^	6.69 ± 0.05^g^	94.51
	LF57	Lighvan cheese	1.5 × 10^7^	7.18 ± 0.02^c^	9.2 × 10^6^	6.96 ± 0.02^f^	97.04
	LF55	Lighvan cheese	7.8 × 10^6^	6.89 ± 0.00 ^de^	4.2 × 10^7^	7.62 ± 0.03^c^	100.00
	D1	Sourdough	1.1 × 10^7^	7.04 ± 0.01 ^cd^	9.3 × 10^6^	6.97 ± 0.11^f^	98.96
	21G	Fermented olives	1.3 × 10^8	8.11 ± 0.00^a^	2 × 10^8	8.30 ± 0.00^a^	100.00
	5G	Camel milk	5.7 × 10^6^	6.76 ± 0.11 ^ef^	4.7 × 10^7^	7.67 ± 0.10^c^	100.00
	61G	Pitcher cheese	5.4 × 10^6^	6.73 ± 0.01 ^ef^	8 × 10^6^	6.90 ± 0.00^f^	100.00
***L. gasseri***	54C	Vaginal	3.6 × 10^7^	7.56 ± 0.02^b^	8.28 × 10^7^	7.92 ± 0.00^b^	100.00
	49A	Vaginal	0.72 × 10^6^	5.86 ± 0.00^h^	0.45 × 10^6^	5.65 ± 0.01^i^	96.41
	47B	Vaginal	1.1 × 10^6^	6.04 ± 0.00^h^	1.7 × 10^7^	7.23 ± 0.00^de^	100.00
	52B	Vaginal	0.95 × 10^6^	5.98 ± 0.01^h^	1.3 × 10^7^	7.11 ± 0.00^e^	100.00
***L. fermentum***	OF	Camel Doogh	3.7 × 10^6^	6.57 ± 0.01 ^ef^	2.1 × 10^7^	7.32 ± 0.01^d^	100.00
	F	ATCC 9338	1.3 × 10^7^	7.11 ± 0.11 ^cd^	1.83 × 10^7^	7.26 ± 0.02^de^	100.00
***L. acidophilus***	AC	ATCC 4356	0.22 × 10^6^	5.34 ± 0.14^i^	4.8 × 10^6^	6.68 ± 0.01^g^	100.00

### 3.3 Tolerance to simulated gastric juice (pepsin)

*L. plantarum* isolated from the small intestine (A7), Lighvan cheese (LF56 and LF57), and fermented olives (21G), and *L. gasseri* isolated from a human source (54C) had a high survival rate in the simulated gastric juice with a pH of 2.5. This experiment had a colony count of less than 10^6^ for *L. gasseri* from human milk (49A, 47 B, and 52 B) and *L. plantarum* from camel milk (5G). Meanwhile, *L. fermentum* from Camel Doogh (OF) and ATCC 9338 (F), *L. acidophilus* ATCC 4356 (AC), *L. plantarum* from Lighvan cheese (LF 55 and LF 48), sourdough (D1), and *L. plantarum* from milk (M8 and M11) from wheat bran (S2G) and Pitcher cheese (61G) showed no colony after treatment in simulated gastric juice (Table 4).

**Table 4:** Survival of LAB strains in simulated gastric condition (pepsin solution (PH = 2.5)).

Species	Strains number	source	Initial counts time (0h) cfu/ml	Log) cfu/ml)	Survival after time (4h) cfu/ml	Log) cfu/ml)	Survival%
***L*. *plantarum***	M8	Milk	>2000	0.00^g^	0.00	0.00^f^	0.00
	M11	Milk	>2000	0.00^g^	0.00	0.00^f^	0.00
	S2G	Wheat bran	0.83 × 10^6^	5.92 ± 0.00^d^	0.00	0.00^f^	0.00
	A7	Small intestine	9.6 × 10^6^	6.98 ± 0.01 ^ab^	1.1 × 10^7^	7.04 ± 0.00^a^	100.00
	LF56	Lighvan cheese	1.2 × 10^6^	6.08 ± 0.02 ^cd^	1.6 × 10^6^	6.20 ± 0.01^c^	100.00
	LF48	Lighvan cheese	1.6 × 10^6^	6.20 ± 0.00 ^cd^	0.00	0.00^f^	0.00
	LF57	Lighvan cheese	0.014 × 10^7^	5.15 ± 0.12^e^	0.93 × 10^6^	5.97 ± 0.02^c^	100.00
	LF55	Lighvan cheese	6.6 × 10^3^	4.11 ± 0.01^f^	0.00	0.00^f^	0.00
	D1	Sourdough	1 × 10^6^	6.00 ± 0.02 ^cd^	0.00	0.00^f^	0.00
	21G	Fermented olives	8 × 10^6^	6.90 ± 0.04 ^ab^	6 × 10^6^	6.78 ± 0.1^b^	98.19
	5G	Camel milk	0.8 × 10^6^	5.90 ± 0.01^d^	0.8 × 10^4^	3.90 ± 0.00^e^	66.1
	61G	Pitcher cheese	1.4 × 104	4.15 ± 0.01^f^	0.00	0.00^f^	0.00
***L*. *gasseri***	54C	Vaginal	0.3 × 10^7^	6.48 ± 0.01 ^bc^	0.1 × 10^7^	6.00 ± 0.01^c^	92.63
	49A	Vaginal	0.2 × 10^5^	4.30 ± 0.00^f^	0.8 × 10^4^	3.90 ± 0.01^e^	90
	47B	Vaginal	5.2 × 10^5^	5.72 ± 0.02^d^	1.2 × 10^4^	4.08 ± 0.00^e^	71
	52B	Vaginal	0.1 × 10^5^	4.00 ± 0.11^f^	0.25 × 10^5^	4.40 ± 0.01^d^	100
***L*. *fermentum***	OF	Camel Doogh	0.6 × 10^6^	5.78 ± 0.14^d^	0.00	0.00^f^	0.00
	F	ATCC 9338	1.3 × 10^4^	3.82 ± 0.04^f^	0.00	0.00^f^	0.00
***L*. *acidophilus***	AC	ATCC 4356	1 × 10^7^	7.00 ± 0.05^a^	0.00	0.00^f^	0.00

### 3.4 Tolerance to simulated intestinal juice (pancreatin)

*L. fermentum* isolated from camel Doogh (OF) and ATCC 9338 (F) and *L. plantarum* originated from Lighvan cheese (LF 56, LF 57, and LF 55) had the maximum survival rate in simulated intestinal juice (pancreatin) with a pH of 8. No colony was observed for *L. plantarum* isolated from milk (M8 and M11) and isolated from pitcher cheese (61G), *L. gasseri* with human source (49A), and *L. acidophilus* ATCC 4356 (AC) on the culture plate after 4 h of treatment (Table 5).

**Table 5:** Survival of LAB strains in simulated intestinal condition (pancreatin) PH = 8)).

Species	Strains number	Source	Initial counts time (0h) cfu/ml	Log) cfu/ml)	Survival after time (4h) cfu/ml	Log) cfu/ml)	Survival%
***L*. *plantarum***	M8	Milk	<10	0.00^i^	0.00	0.00^g^	0.00
	M11	Milk	0.84 × 10^4^	3.92 ± 0.00^h^	0.00	0.00^g^	0.00
	S2G	Wheat bran	1.1 × 10^8^	8.04 ± 0.05^a^	0.18 × 10^7^	6.26 ± 0.01 ^cd^	77.79
	A7	Small intestine	0.1 × 10^7^	6.00 ± 0.07^f^	0.6 × 10^6^	5.78 ± 0.00 ^ef^	96.30
	LF56	Lighvan cheese	0.23 × 10^6^	5.36 ± 0.01^g^	0.11 × 10^7^	6.04 ± 0.00 ^de^	100.00
	LF48	Lighvan cheese	0.53 × 10^7^	6.72 ± 0.00 ^cd^	0.36 × 10^7^	6.56 ± 0.03 ^bc^	97.50
	LF57	Lighvan cheese	0.38 × 10^7^	6.58 ± 0.00^cde^	0.81 × 10^7^	6.91 ± 0.00^b^	100.00
	LF55	Lighvan cheese	0.16 × 10^6^	5.20 ± 0.06^g^	0.4 × 10^6^	5.60 ± 0.00^f^	100.00
	D1	Sourdough	0.9 × 10^7^	6.95 ± 0.07^c^	0.43 × 10^7^	6.63 ± 0.02^b^	95.39
	21G	Fermented olives	0.11 × 10^8^	7.04 ± 0.14 ^bc^	0.15 × 10^7^	6.18 ± 0.01^d^	87.71
	5G	Camel milk	0.36 × 10^7^	6.56 ± 0.01^cde^	0.11 × 10^7^	6.04 ± 0.00 ^de^	92.15
	61G	Pitcher cheese	0.11 × 10^6^	5.04 ± 0.04^g^	0.00	0.00^g^	0.00
***L*. *gasseri***	54C	Vaginal	0.3 × 10^7^	6.48 ± 0.10^def^	0.1 × 10^7^	6.00 ± 0.16 ^de^	92.63
	49A	Vaginal	0.014 × 10^6^	4.15 ± 0.05^h^	0.00	0.00^g^	0.00
	47B	Vaginal	0.12 × 10^7^	6.08 ± 0.03 ^ef^	0.21 × 10^6^	6.00 ± 0.01^de^	98.70
	52B	Vaginal	0.1 × 10^7^	6.00 ± 0.11^f^	0.5 × 10^6^	5.70 ± 0.00 ^ef^	94.98
***L*. *fermentum***	OF	Camel Doogh	0.27 × 10^8^	7.43 ± 0.08^b^	0.8 × 10^8^	7.90 ± 0.00^a^	100.00
	F	ATCC 9338	0.4 × 10^7^	6.60 ± 0.00^cde^	0.63 × 10^7^	6.80 ± 0.00^b^	100.00
***L*. *acidophilus***	AC	ATCC 4356	0.94 × 10^7^	6.97 ± 0.00 ^cd^	0.00	0.00^g^	0.00

### 3.5 Surface hydrophobicity properties of LAB strains

**Fig. 1:**
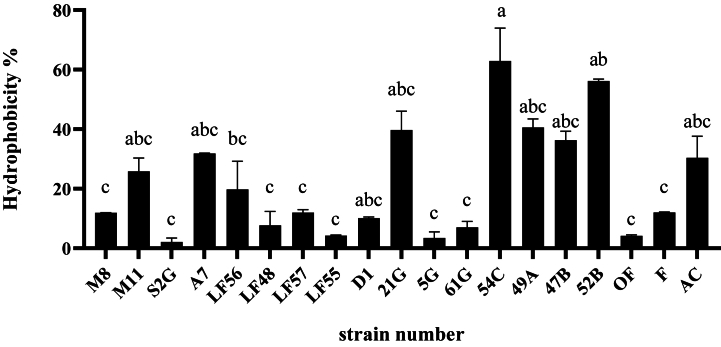
Surface hydrophobicity properties of LAB strains. Different letters indicate significant differences (P < 0.05).

In the present study, the strain with the highest degree of hydrophobicity was L. gasseri isolated from a human source, exhibiting a surface hydrophobicity of 56.56 %. Also, *L. acidophilus* ATCC 4356 (AC), *L. plantarum* isolated from the small intestine (A7), and *L. plantarum* isolated from fermented olive (21G) had moderate adhesions of 31.80, 30.38, and 39.69 %, respectively. The majority of the strains had weak hydrophobicity (Fig. 1).

### 3.6 Antibiotic resistance test

Different strains of *L. plantarum* were sensitive to the tested antibiotics and resistant to kanamycin and chloramphenicol. Also, M11, S2G, A7, LF55, LF57, and 5G were resistant to chloramphenicol and M8, M11, and 61G to kanamycin. *L. gasseri* showed moderate resistance to ampicillin, erythromycin, and tetracycline, while *L. fermentum* ATCC 9338 (F) showed good resistance to ampicillin, erythromycin, and chloramphenicol. The highest antibiotic resistance for *L. acidophilus* ATCC 4356 (AC) is related to ampicillin, chloramphenicol, and tetracycline (Table 6).

**Table 6:** Minimum inhibitory concentration (MIC, mg/l) of 5 antibiotics on LAB strains.

Species	Strains number	source	Ampicillin	kanamycin	Erythromycin	chloramphenicol	tetracycline
***L.plantarum***	M8	Milk	0.9 ± 0.14^f^	1.4 ± 0.002^b^	0.5 ± 0.00^a^	0.007 ± 0^b^	0.375 ± 0.3 ^ab^
	M11	Milk	0.25 ± 0.00^de^	1.1 ± 0.14^b^	0.5 ± 0.00^a^	ND^b^	0.031 ± 0.00^ef^
	S2G	Wheat bran	2 ± 0.37^b^	0.25 ± 0.00^b^	0.25 ± 0.00 ^bc^	ND^b^	0.25 ± 0.00 ^cd^
	A7	Small intestine	1.2 ± 0.31^bc^	0.12 ± 0.00^b^	0.125 ± 0.00 ^cd^	ND^b^	0.5 ± 0.00^a^
	LF56	Lighvan cheese	0.9 ± 0.28 ^cd^	0.5 ± 0.00^a^	0.5 ± 0.00^a^	0.5 ± 0.00^a^	1 ± 0.0175 ^ef^
	LF48	Lighvan cheese	0.25 ± 0.00^de^	0.5 ± 0.00^a^	0.25 ± 0.00 ^bc^	0.25 ± 0.00^b^	0.5 ± 0.00^a^
	LF57	Lighvan cheese	0.03 ± 0.00^g^	0.5 ± 0.00^a^	0.31 ± 0.085 ^ab^	ND^b^	0.125 ± 0.00^def^
	LF55	Lighvan cheese	0.001 ± 0.00^g^	0.5 ± 0.00^a^	0.25 ± 0.00 ^bc^	ND^b^	0.015 ± 0.00^f^
	D1	Sourdough	0.03 ± 0.00^g^	0.5 ± 0.00^a^	0.25 ± 0.00 ^bc^	0.001 ± 0.00^b^	0.125 ± 0.00^def^
	21G	Fermented olives	2.1 ± 0.37^b^	0.5 ± 0.00^a^	0.5 ± 0.00 a	0.5 ± 0.00^a^	0.5 ± 0.00^a^
	5G	Camel milk	0.12 ± 0.00^f^	0.12 ± 0.00^b^	0.125 ± 0.00 ^cd^	ND^b^	0.25 ± 0.00 ^cd^
	61G	Pitcher cheese	0.8 ± 0.13^f^	1.3 ± 0.002^b^	0.062 ± 0.00^d^	0.003 ± 0.00^b^	0.0024 ± 0.00^f^
***L.gasseri***	54C	Vaginal	0.9 ± 0.12^f^	0.5 ± 0.00^a^	0.25 ± 0.00 ^bc^	ND^b^	0.015 ± 0.00^f^
	49A	Vaginal	0.015 ± 0.00^g^	0.5 ± 0.00^a^	0.015 ± 0.00^d^	0.5 ± 0.00^a^	0.031 ± 0.00^ef^
	47B	Vaginal	0.9 ± 0.28 ^cd^	0.5 ± 0.00^a^	0.5 ± 0.00^a^	ND^b^	1.2 ± 0.2655^bc^
	52B	Vaginal	0.5 ± 0.00^a^	0.5 ± 0.00^a^	0.125 ± 0.00 ^cd^	ND^b^	0.062 ± 0.00^ef^
***L.fermentum***	OF	Camel Doogh	2.00 ± 0.020^g^	0.5 ± 0.00^a^	1.13 ± 0.005^d^	ND^b^	0.25 ± 0.00 ^cd^
	F	ATCC 9338	0.9 ± 0.004^g^	0.25 ± 0.00^b^	0.94 ± 0.009^d^	ND^b^	0.125 ± 0.00^def^
***L.acidophilus***	AC	ATCC 4356	2.1 ± 0.19 ^ef^	0.03 ± 0.00^b^	0.25 ± 0.00 ^bc^	ND^b^	1.00 ± 0.1405^cde^

ND: Not determined.

### 3.7 Auto-aggregation and Co-aggregation abilities of LAB strains

**Fig. 2:**
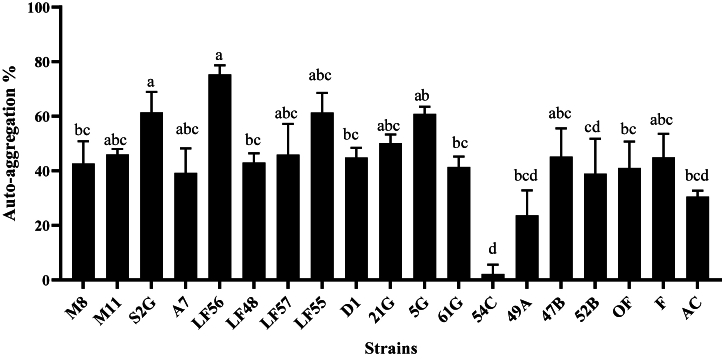
Percentages of auto-aggregation abilities of LAB strains.

**Fig. 3:**
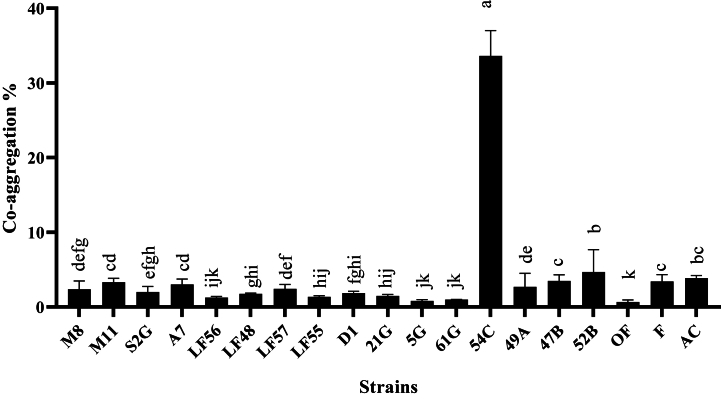
Comparison of co-aggregation abilities percentages among different LAB strains and *Salmonella enterica*.

**Fig. 4:**
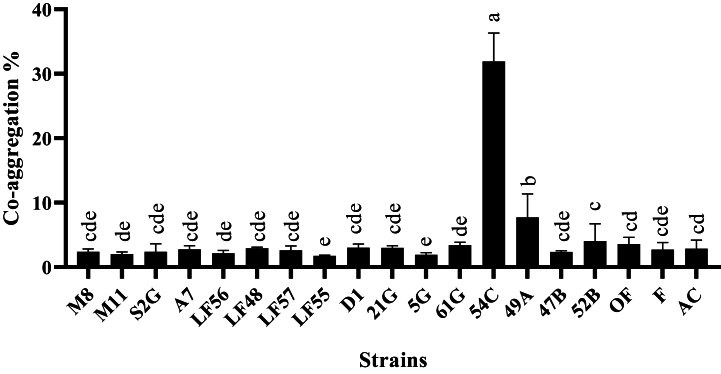
Comparison of co-aggregation abilities percentages among different LAB strains and *Escherichia coli*.

**Fig. 5:**
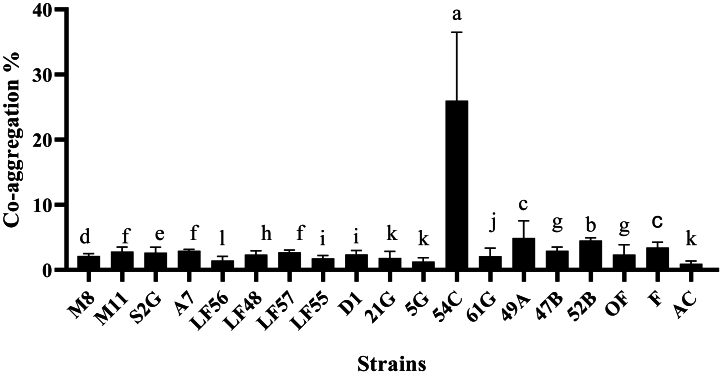
Comparison of co-aggregation abilities percentages among different LAB strains and *Listeria monocytogenes.*

In the auto-aggregation test, all strains had moderate sedimentation, except for *L. gasseri* (54C), isolated from human sources, which showed weak sedimentation. The highest auto-aggregation percentage was related to *L. plantarum* isolated from Ligvan cheese with code LF56 at the rate of 75 % (Fig. 2). Also, in the co-aggregation test with the pathogenic bacteria *E. coli* O157:H7 NCTC 12900, *S. enterica* subsp*. enterica* ATCC 13076, and *L. monocytogenes* ATTC 7644, the highest precipitation was related to the *L. gasseri* strain with code 54C on *S. enterica* subsp*. enterica* ATCC 13076 by 33.63 *%* (Fig. 3)*, E. coli* by 31.93 % (Fig. 4), and *L. monocytogenes* by 24.98 % (Fig. 5). The sedimentation of the rest of the strains was almost at the same level [23].

### 3.8 The amount of short-chain fatty acids produced by *L. fermentum* and *L. acidophilus*

**Fig. 6:**
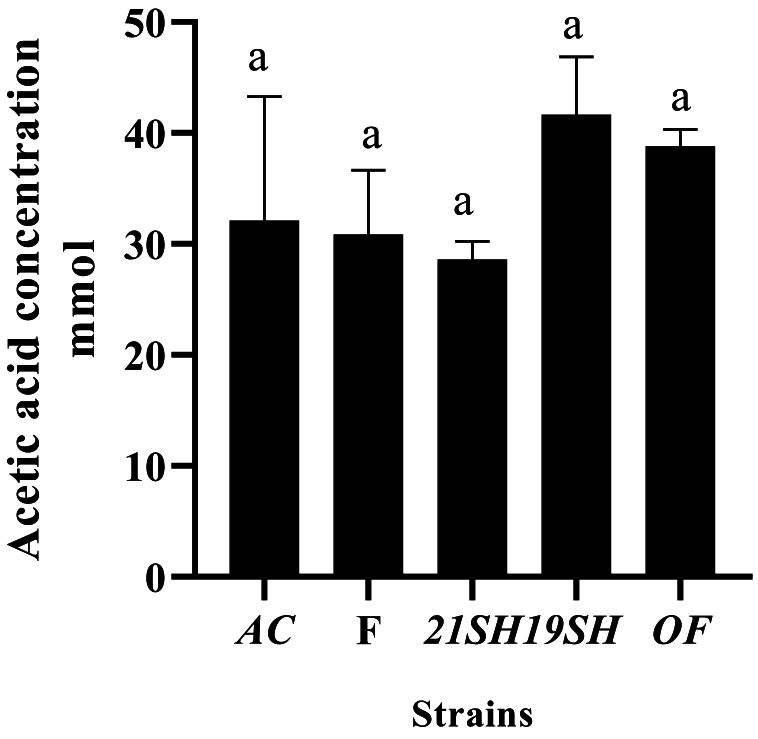
Production of acetic acid by *Limosilactobacillus fermentum* (F- ATCC 9338, 19SH, 21SH -ATCC 14931, OF) and *Lactobacillus acidophilus* AC (ATCC 4356).

**Fig. 7:**
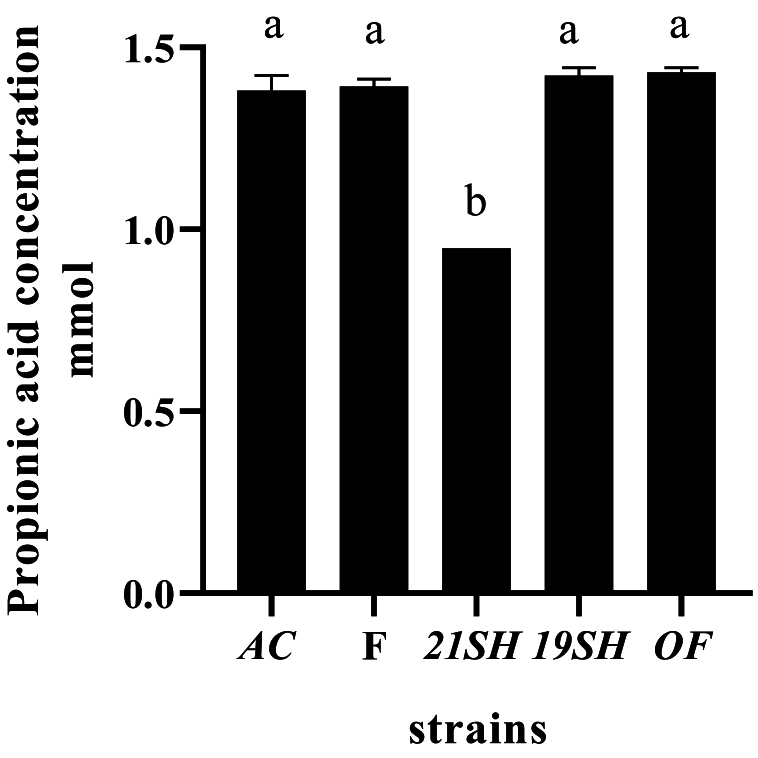
Production of propionic acid by *Limosilactobacillus fermentum* (F- ATCC 9338, 19SH, 21SH -ATCC 14931, OF) and *Lactobacillus acidophilus* AC (ATCC 4356).

**Fig. 8:**
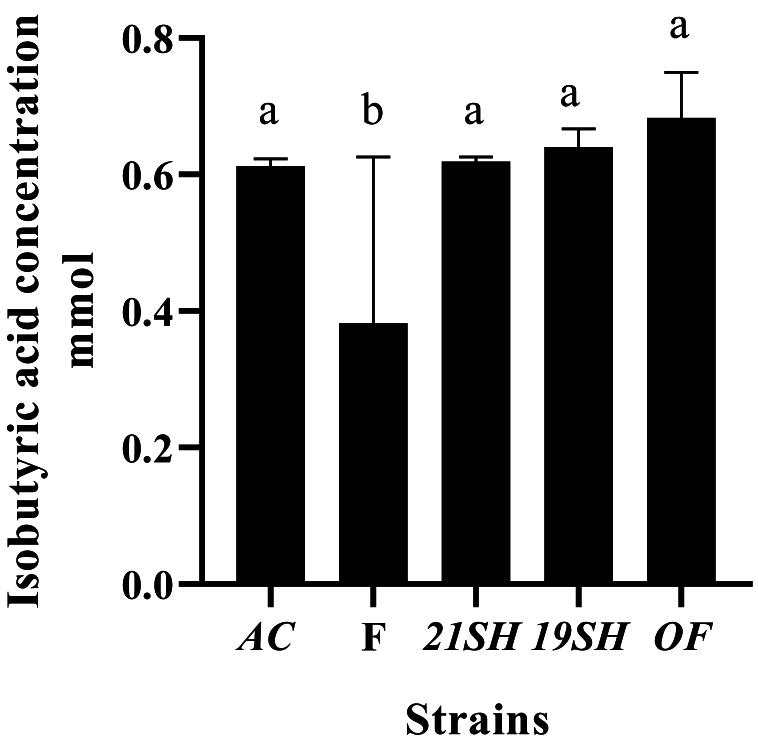
Production of isobutyric acid by *Limosilactobacillus fermentum* (F- ATCC 9338, 19SH, 21SH -ATCC 14931, OF) and *Lactobacillus acidophilus* AC (ATCC 4356).

**Fig. 9:**
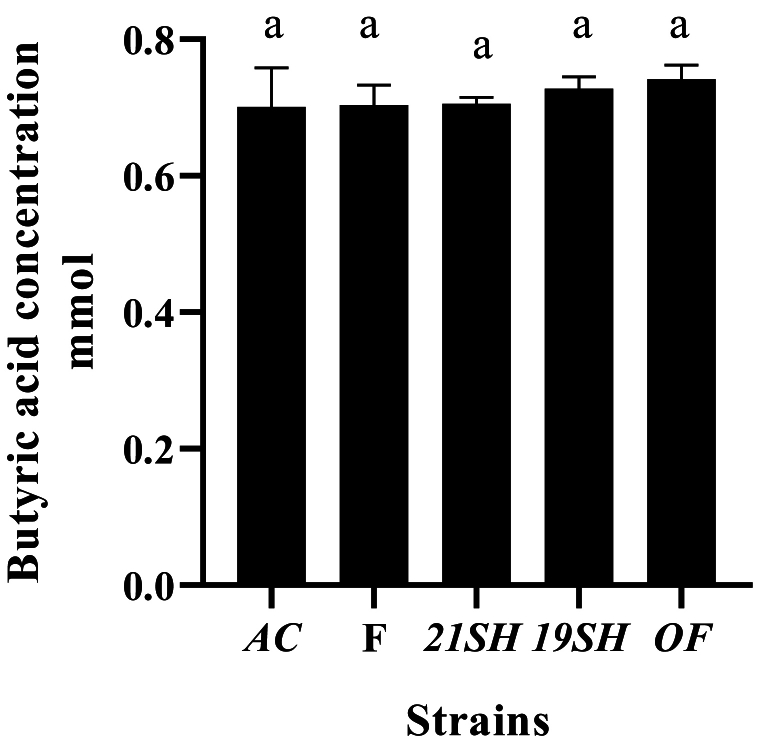
Production of butyric acid by *Limosilactobacillus fermentum* (F- ATCC 9338, 19SH, 21SH -ATCC 14931, OF) and *Lactobacillus acidophilus* AC (ATCC 4356).

**Fig. 10:**
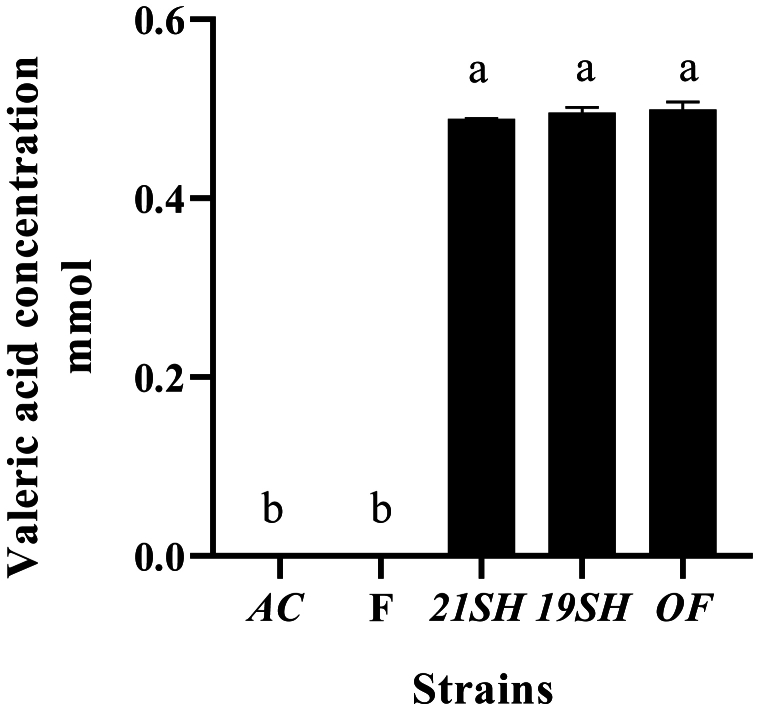
Production of valeric acid by *Limosilactobacillus fermentum* (F- ATCC 9338, 19SH, 21SH -ATCC 14931, OF) and *Lactobacillus acidophilus* AC (ATCC 4356).

**Fig. 11:**
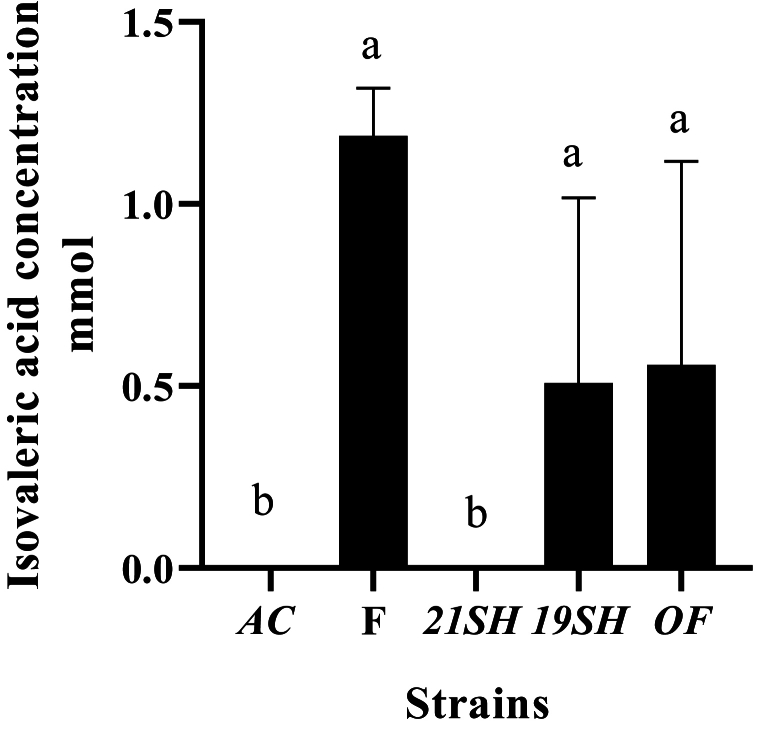
Production of isovaleric acid by *Limosilactobacillus fermentum* (F- ATCC 9338, 19SH, 21SH -ATCC 14931, OF) and *Lactobacillus acidophilus* AC (ATCC 4356).

GC-FID was performed to determine the amounts of short-chain fatty acids produced by *L. fermentum* and *L. acidophilus* strains, including acetic acid, propionic acid, butyric acid, *iso*-butyric acid, valeric acid, and *iso*-valeric acid. Acetic acid production was related to *L. acidophilus* and *fermentum* strains as the highest and the lowest amounts were produced by *L. fermentum* 19SH (41.62 mmol) and *L. fermentum* (21SH-ATCC 14931) (28.68 mmol), respectively (Fig. 6). According to Fig. 7, the highest amount of propionic acid is related to *L. fermentum* (OF) and (19SH) with 1.43 and 1.42 mmol, respectively. In contrast, the lowest amount was related to *L. fermentum* (21SH) (0.94 mmol) (Fig. 7). Also, the highest amount of *iso*-butyric acid production is related to *L. fermentum* (OF) with 0.67 mmol and the lowest amount is related to *L. fermentum* (F) with 0.38 mmol (Fig. 8). The highest amount of butyric acid is related to *L. fermentum* (OF) and *L. fermentum* (19SH) with 0.76 mmol and 0.74 mmol, respectively. *L. fermentum* (21SH and F) and *L. acidophilus* (AC) produced the same amount of butyric acid (i.e., 0.7 mmol) (Fig. 9). The highest amount of valeric acid production is related to *L. fermentum* (OF and 19SH) with 0.49 mmol and 0.48 mmol respectively. *L. fermentum* (F) and *L. acidophilus* (AC) were not productive (Fig. 10). According to Fig. 11, the highest amount of *iso*-valeric acid is produced by *L. fermentum* (F) with 1.18 mmol. *L. fermentum* (21SH) and *L. acidophilus* (AC) do not produce *iso*-valeric acid (Fig. 11).

### 3.9 Induction of IL-12 in mouse splenocytes by *stimulation of L. plantarum* and *L. gasseri strains*

**Fig. 12:**
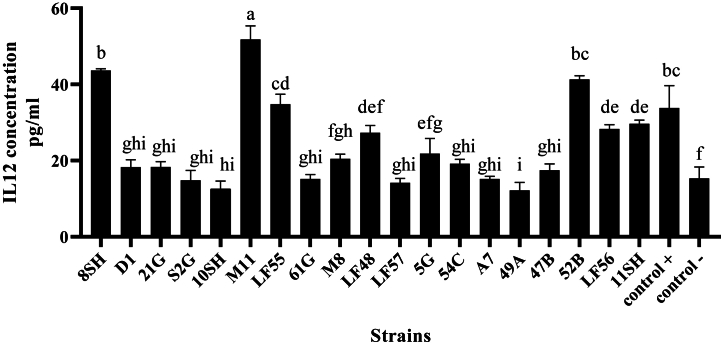
Induction of interleukin 12(p70) by *Lactiplantibacillus plantarum* (LF48, LF55, LF56, LF57, M8, M11, S2G, A7, D1, 5G, 21G, 8SH-ATCC14917, 10SH, 11SH, 61G) and *Lactobacillus gasseri* (52B, 49A, 47B, 54C).

Among L. *plantarum* strains, the highest and the lowest inductions of IL-12 (p70) were observed in *L. plantarum* (M11; 51.83 pg/mL) and (10SH; 12.5 pg/mL), respectively. Meanwhile, the highest and the lowest inductions of IL-12 (p70) among *L. gasseri* strains were related to 52B (41.33 pg/mL) and 49A (12.16 pg/mL), respectively (Fig. 12). Among L. *plantarum* strains, the highest inductions of IL-12 (p70) were related to raw milk (M11) and Sauerkraut (8SH). Meanwhile, the lowest inductions of IL-12 (p70) were isolated from Tarkhine (10SH) and Lighvan cheese (LF57) (Fig. 12). In general, *L. plantarum* strains induced more IL-12 (p70) than other strains (Fig. 12).

## 4 Discussion

According to the FAO/WHO criteria for assessing probiotic properties, probiotic microorganisms should not only survive passage across the digestive tract but also proliferate in the intestine. This requirement guarantees their immunity to gastric juices and the ability to grow in the presence of bile under intestinal conditions or be consumed. Probiotic bacteria have antimicrobial activity against human pathogens and can bind to human mucosal cells [24]. A study on probiotic properties reported that all strains of *L. plantarum, acidophilus, fermentum*, and *gasseri* have a strong tolerance to acidic conditions at a pH of 2.5. The strains of *L. plantarum* isolated from milk (M8) and (LF56 and LF57) from Lighvan cheese, and the strain of *L. gasseri* isolated from the vagina of a healthy woman (54C) had the best resistance to acid or low pH as their survival was 100 %. Acids in the human intestine (e.g., hydrochloric acid) are highly oxidizing and dissolve cell biomolecules such as fatty acids, proteins, and DNA molecules. Lowering the pH of the environment prevents metabolism and decreases the growth and survival of lactic acid bacteria [25]. Tolerance to the acidic conditions of these bacteria is related to their inherent properties. The explanation is that *L. plantarum* is a heterofermentative bacterium that can ferment raffinose polysaccharides and other carbohydrates, leading to its higher acid tolerance than other lactobacilli. Also, *L. gasseri* is widely found in the human intestine and gastrointestinal tract, especially in breast milk, and can easily withstand the stomach's acidic environment [[26], [27], [28]].

In addition, the majority of 19 strains had 100 % viability in the synthetic bile salt of pancreatic juice, except for *L. plantarum* isolated from Lighvan cheese (LF56). In this respect, there is a comparatively high quantity of bile salt in the small and large intestines, which is poisonous and harmful to living organisms. Therefore, resistance and stability in the presence of bile salts are among the most important characteristics of probiotic bacteria to consider when choosing them [29]. As bacteria are exposed to bile salts, their cellular homeostasis is disrupted, and the degradation of lipid membranes and cell membrane proteins results in bacterial material damage and cell death [30]. Some strains’ resistance to bile salts is related to bile salt hydrolysis activity, as bile salt hydrolysis lowers toxicity and side effects of bile salt [31].

In the present study, the survival rates of strains A7, LF56, and LF57 in simulated gastric juice were 100 %, indicating the strain-specific ability to withstand the acidic conditions of the gastric environment. This result highlights the importance of strain selection in determining the efficacy of probiotic survival in the gastrointestinal tract. Previous research has also emphasized the significance of strain-specific characteristics in probiotic functionality and survival in the human digestive system [32]. Even certain foods can help environmental bacteria survive in the human body. However, these factors are not considered in our simulated model of the gastrointestinal tract. According to the literature, *Lactobacillus* tolerance to gastric juice depends on the activity of the ATPaz hydrogen pump and the composition of bacterial membranes. Furthermore, it depends on the bacteria, the culture medium, and the incubation conditions [33].

Another excellent aspect of probiotic bacteria is their ability to colonize the intestinal wall. As a result, accessibility to the intestinal epithelium (also known as a precondition for colonization) is a critical indicator and requirement when selecting probiotic bacteria [34]. *L. gasseri* can be isolated from intestinal tissue, oral cavity, vaginal region, urine, and human blood, suggesting the strong bond of this bacterium with the human body. Regarding the difference in the amount of D-alanine and the proportion of hexose group in the cell wall liposuction anchor relative to other sources, this strain has the highest ability to bind to intestinal epithelial cells [35]. The results of the colonization of bacteria (especially *L. gasseri)* in this study are consistent with those of previous studies.

Co-aggregation is a highly efficient probiotic process that inhibits pathogens from adhering to the surface of intestinal epithelial cells [36]. The ability of the probiotic bacteria to interact with pathogens and competition with them for adhesion to the intestinal epithelial cell surface has been significantly proven. This dependence is due to the presence of molecules on the membrane surface of probiotic strains of lactic acid bacteria that act as ligands to bind to the pathogens. Consistent with the findings of the previous studies, the human strain of *L. gasseri* had the highest adhesion and cohesive properties [20].

Several strains of *L. plantarum* were sensitive to the studied antibiotic substance. However, M8 and 21G were resistant to kanamycin and ampicillin, respectively, and M11, S2G, A7, LF55, 5G, and LF57 showed high resistance against chloramphenicol. In addition, *L. gasseri* had medium resistance to ampicillin, erythromycin, and tetracycline, while *L. fermentum* showed high resistance to chloramphenicol and ampicillin.

Some probiotic bacteria resist various antibiotics such as vancomycin, streptomycin, gentamicin, and ciprofloxacin. The explanation is that the factors effective in antibiotic resistance (e.g., antibiotic-resistant genes) can be transferred from one lactobacillus to another and, most importantly, to pathogenic bacteria such as staphylococcus [37].

Prior to applying these probiotic bacteria, more research is required to comprehensively assess the profiles of their antibiotic resistance as well as their ability to transfer resistance genes to pathogens. Further studies should also be conducted on the screening of the genotypic and phenotypic characteristics of antibiotic resistance, particularly against classes concentrate critical antibiotics such as aminoglycosides, cephalosporins, and quinolones, while lacking transferable resistance genes such as parC, aac(6′) Ii, ermB, ermC, and tetM. It is necessary to understand and mitigate the antibiotic resistance of probiotic bacteria to ensure that they are effective and safe for use in food and pharmaceutical applications, guaranteeing comprehensive investigation prior to extensive implementation [38].

Joghatai et al. [39] examined the probiotic properties of *L. plantarum*, *fermentum*, and *acidophilus* isolated from fermented and human sources. In this research, the *L. fermentum* isolated from Horre (19SH) had the highest survival rate in the simulated environment with gastric acid (96 %) and co-aggregation with S. enterica subsp.enterica serovar *typhimurium* (51 %). Moreover, *L. fermentum* (OF) isolated from traditionally fermented foods was among the strains with the highest survival in acidic environments.

Since the highest amount of fatty acid produced was from *L. fermentum*, they might have differences in the cell wall. Cell wall thickness increases during short-chain fatty acid production in the logarithmic phase, which varies among strains. In this research, 5 strains of probiotic bacteria from human, fermentative, and dairy sources produced short-chain fatty acids such as acetic acid, propionic acid, butyric acid, isobutyric acid, valeric acid, and isovaleric acid. L. fermentum (19 SH) isolated from Horre (traditional-fermented food) produced the highest amount of acetic acid as short-chain fatty acids. Besides, the maximum quantities of propionic, isobutyric, butyric, and valeric acid were produced by *L. fermentum* (OF) isolated from camel doogh (traditional-fermented food). *L. fermentum* (ATCC 9338) (F) generated the maximum level of producing isovaleric acid from a human source. The highest volume of fatty acids released by *L. fermentum* was due to a difference in the cell wall between *L. fermentum* and *acidophilus.* Tanigawa and Umezu [40] compared the cell wall thickness of *L. fermentum* H-34, *L. heterohiochii* H-1, *Leuconostoc mesenteroides* (IFO3832), and *Latilactobacillus sakei* (IFO3541) isolated from fermented foods. The results revealed that *L. fermentum* (H-34) and *L. heterohiochii* (H-1) had thicker cell walls than the control bacteria (i.e., 600 Å versus 200 Å for the control bacteria). In addition, findings showed that wall thickness increases with increasing bacterial growth as a function of the bacterial growth period [40]. A similar study investigated fatty acids formed by L. fermentum (NCIMB 5221) and L. acidophilus (RD758) isolated from fermented foods. The results showed that *L. fermentum* (NCIMB 5221) was able to produce ferulic acid (FA) as an antioxidant and antitumor compound. Moreover, they produced free fatty acids (FFA), thereby inhibiting the growth of colon cancer cells [7,8]. Acetic acid is the most prevalent short-chain fatty acid in the large intestine [6]. In this research, most of the acetic acid was produced by *L. fermentum* (19SH). Due to the large intestine's high amount of acetic acid, more than half of all short-chain fatty acids in the feces are acetic acid [41]. The acetic acid concentration in the large intestine exceeds that of all short-chain fatty acids in cells, making it a key component in carbohydrate and fat metabolism [42]. One of the most important effects of short-chain fatty acids is that they lower pH, thereby lowering the population of pathogenic microorganisms and improving nutrient absorption. In addition, a compound like a butyrate can modify intestinal epithelial cells and increase mucus production, which helps bacteria adhere better [43]. Research has shown that *L. fermentum* (NCIMB 5221) prevents the development of colon cancer cells by producing short-chain fatty acids while promoting the growth of intestinal epithelial cells. *Lactobacillus* proliferation is highly effective in the proliferation of cancer cells, thereby damaging cancer cells by preventing their proliferation. Besides, they are beneficial to normal colon cells. This effect is strongly linked to these bacteria’ ability to produce more short-chain fatty acids, especially acetic, butyric, and propionic acid, compared to other probiotic bacteria [7]. Among SCFAs, butyric acid has the highest anti-inflammatory activity. The energy loss originates from the intestinal mucosa's inflammatory phase, which appears in combination with multiple pathological processes. In this regard, butyric acid is the primary energy source for intestinal epithelial cells, wherein butyric acid has a beneficial immune system regulating effect on intestinal epithelial cells and other mucosal cells. Butyrate induces apoptosis in colorectal cancer cells and lymphomas [44,45]. Butyrate also inhibits the growth and development of colon stem cells, which are found at the end of the intestine. Several human studies have shown significantly lower butyrate levels in patients with colorectal cancer than in healthy people [46]. In the present study, the *L. fermentum* strain from camel doogh produced the most butyric acid (0.74 mmol). In contrast to other short-chain fatty acids, the role of valeric acid in the gut is unclear. Valeric acid has been shown in a few experiments to promote the development of intestinal epithelium and to be active in diseases including colitis, heart-metabolic diseases, and cancer [47,48]. The strain of *L. fermentum* (ATCC 9338) (F) from a human produced the most isovaleric acid (1.18 mmol) in this study. Propionic acid is primarily formed by Gram-positive bacteria in the human intestine [49]. In the liver, this acid inhibits gluconeogenesis and cholesterol synthesis [50]. It also has antibacterial, anti-inflammatory, and antitumor properties, thereby protecting the human gut from microbial pathogens [51,52]. In this study, *L. fermentum* (OF) isolated from camel doogh provided the most propionic acid (1.43 mmol). In previous research, prescriptions for lactic acid bacteria and fermented dairy goods have been found to affect the activities of digestive enzymes related to intestinal cancer compounds. Several intestinal bacteria can transform non-toxic compounds into metabolites that cause tumorigenesis and inflammation [53]. Since probiotic bacteria contain lactic and acetic acids, they lower intestinal pH and establish an atmosphere conducive to removing intestinal microbial pathogens and modifying bacterial enzymes [54]. In this respect, previous studies have shown that administering probiotic bacteria such as *L. acidophilus* to patients with colon cancer reduced fecal pH, decreased infectious bacteria, and increased antitumor and anticancer effects significantly [55].

According to the ELISA results, the highest induction of IL-12 in the culture of lymphocyte cells was related to *L. plantarum* (8SH) isolated from Sauerkraut, a traditionally fermented food, *L. plantarum* (M11) isolated from milk, and *L. gasseri* (52B) isolated from the vagina. On the other hand, the lowest amount is related to *L. plantarum* (10SH) isolated from Tarkhineh (12.5), *L. plantarum* (LF57) isolated from Lighvan cheese (14.16), and *L. gasseri* (49A) isolated from the vagina (12.16). More adhesion to the intestine's inner wall or more colonization in the intestine is among the important factors for inducing IL-12 in mouse splenocytes by bacteria. This factor can be estimated using amounts of adhesion, auto-aggregation, and co-aggregation of bacteria.

According to Joghataei et al. [39], *L. plantarum* (8SH) isolated from Sauerkraut (due to more tendency to adhesion or colonization greatly) decreases the adhesion of *Escherichia coli* bacteria to HT-29 cells. Also, this bacterium inhibited the development of *E. coli* O157: H7 and S. enterica subsp.enterica serovar *typhimurium* (ATCC 14028). The hydrophobicity level of this bacterium was 33 %, while its auto-aggregation and co-aggregation with *E. coli* and S. enterica subsp.enterica serovar *typhimurium* was reported as 51 %, 31 %, and 41 %, respectively. According to this study, the hydrophobicity levels of *L. plantarum* (M11) and *L. gasseri* (52B) were 25 % and 57 %, respectively. Also, the result of auto-aggregation was 46 % and 40 %, respectively, and the rate of co-aggregation on the three pathogens studied was about 5 %. The present study showed that *L. gasseri* (52B) has the highest adhesion level, with the same amount of auto-aggregation. Meanwhile, *L. plantarm* (8SH) had the highest co-aggregation. The ability of both probiotic and lactic acid strains to interact with pathogens and compete for adherence to the intestinal epithelial cell surface was consistent with those reported in similar studies. Since the aggressive potential of the pathogenic bacteria seems to be related to the ability to bind to epithelial cells, the impact of auto-aggregation and co-aggregation is very significant for probiotic bacteria. This factor can be estimated using amounts of adhesion, auto-aggregation, and co-aggregation of bacteria. This dependence can be due to the presence of certain molecules on the membrane surface of probiotic lactic acid bacteria strains that function as ligands and bind to pathogens or in the position of antibiotic resistance. Lactic acid bacteria strains are bound to the intestinal epithelial cells by a bandage [20]. However, among these three strains, *L. plantarum* (M11) had the highest rate of IL-12 induction, which is typically related to its cell wall. Murasaki et al. [12] investigated the stimulation of IL-12 by L*. plantarum* L-137 cell wall lipoteichoic acid compared to *L. plantarum* JCM1149. According to their results, the lipoteichoic acid genes synthesized from *L. plantarum* L-137's cell wall induced more IL-12 (p40) in mouse splenocytes and dendritic cells than in the control group. Cell wall components in gram-positive bacteria (e.g., lipoteichoic acid and non-methyl peptidoglycans) also stimulate immune cells. According to these researchers, the compound had no additive effect on the induction of IL-12 (p40) in their experiments on peptidoglycans. TLR2 receptors initiate signals in response to molecular patterns associated with pathogens and inflammatory agents. These receptors are stimulated by *L. plantarum* L137 lipoteichoic acid, but peptidoglycans are not altered [12]. Kaji et al. [56] compared lipoteichoic acid in *Lacticaseibacillus casei*, *L*.*plantarum,* and *L*.*gasser*i bacteria in terms of IL-10 and IL-12 induction. According to these authors, in lactic acid bacteria, the teichoic acid of the cell wall is spread from peptidoglycan and lipoteichoic acid from the plasma membrane. It has been determined that the lipoteichoic acid of *L. plantarum* has an incremental effect on IL-12 induction. Grangette et al. [57] investigated the immune system and the induction of inflammatory cytokines such as IL-10 and IL-12 by L*. plantarum* NCIMB8826 cell wall lipoteichoic acid. These researchers reported that cell wall mutations that affect lipoteichoic acid D-alanylation (reduction of D-alanine lipoteichoic acid and glucose replacement) result in a sharp increase in IL-10 induction [57]. The cell wall of probiotic bacteria plays a significant role in how well they adhere to free intestinal toxins. According to previous studies, the structure of probiotic bacteria is associated with a decrease in tumorigenesis [58]. Lactobacilli cellular components (e.g., entire cells, heat-inactivated cells, cell walls, peptidoglycans, and cytoplasmic extracts) have various functions when exposed to cancer cells [59]. Reiki et al. [60] found that *Lactobacillus* cytoplasmic extract and cell wall decreased the protein level of the P53 mutant gene significantly. The P53 gene is a tumor suppressor gene that plays an important role in cell apoptosis. This gene acts as a guard for the cell and inhibits the replication process in the damaged DNA of the cell. Probiotic bacteria, like lactobacilli, inhibit many pathological cell types. This type of bacteria strengthens the host's immune system and helps prevent cancer by inhibiting the expression of malignant tumors [31]. Increased levels of cytokines and immunoglobulins, increased mononucleate cell proliferation, activation of natural macrophages, and inhibitory macrophages are all effects of probiotic bacteria on the immune system. The stimulation of immunity against pathogenic bacteria and protozoa is referred to as autoimmunity. T cells' cytokine production and lymphocyte differentiation are also suppressed by probiotic bacteria. All bacteria have been shown to stimulate immune cell proliferation and the synthesis of pro-inflammatory cytokines, including TNF-α and IL-12 [61,62]. Most significantly, probiotic bacteria have these beneficial effects on the immune system without causing an inflammatory response that is harmful to the body. The immune response can be enhanced when many probiotic bacteria are taken together and function synergistically. This result is most common when Lactobacillus and Bifidobacteria are consumed together.

## 5 Conclusion

Lactic acid bacteria strains with a high potential to adhere to intestinal epithelial cells colonize the intestine more effectively than other strains. These strains function as pathogen inhibition using various mechanisms, including antimicrobial activity, co-aggregation with pathogens, and attachment to intestinal epithelial cells. *L. gasseri* strain isolated from the vagina (54C) is the strongest probiotic strain among the lactic acid bacteria examined in this study. They have a significant level of antimicrobial activity and a strong capacity to prevent pathogen infection through co-aggregation. Different variations of lactic acid bacterial strains may also inhibit pathogen invasion, confirming the importance of creating new and specialized probiotic compounds to prevent infections and the host's health. IL-12 plays an essential role in controlling the function of probiotic compounds, improving the response of Th1 cells, and regulating cellular immune activity in particular. Therefore, the potential of a lactobacillus strain to induce IL-12 induction may be a useful measure of immune stimulatory operation. The composition of lactobacilli's cell walls is a key component in inducing IL-12 release and effectively stimulates macrophages and dendritic cells to metabolize IL-12. *L. plantarum* isolated from Sauerkraut (8SH), *L. plantarum* isolated from milk (M11), and *L. gasseri* isolated from the vagina (52B) showed the maximum induction of IL-12. Also, it is also predicted that *L. plantarum* isolated from milk and *L. gasseri* from the vagina has beneficial immunological properties due to the presence of strong adhesion genes. Factors such as microbial growth curve and choosing the right extraction method are effective in producing short-chain fatty acids as antitumor compounds. *L. fermentum* (19SH) and (OF) isolated from traditional fermented foods are a viable alternative owing to their high development of antitumor compounds, including acetic acid, propionic acid, butyric acid, isobutyric acid, valeric acid, and isovaleric acid. This effect is mostly attributed to its thicker cell wall than *L. acidophilus* (ATCC 4356) (AC). The ability of probiotic bacteria strains to survive in the gastrointestinal tract is an exciting and significant feature, most probably regarding their cell wall strength and stiffness.

## Funding sources

This work was supported by a grant (No. 3/48972) from by the research deputy of 10.13039/501100003121Ferdowsi University of Mashhad, Iran.

## Ethics approval

All applicable international, national, and/or institutional guidelines for the care and use of animals were followed. This study involves animal testing (mice). The ethical criteria with the code of IR.UM.REC.1400.004 (Ferdowsi University of Mashhad, Iran) were observed.

## Data availability statements

All data generated or analyzed during this study are included in this published article.

## CRediT authorship contribution statement

**Parinaz Mobasherpour:** Writing – original draft, Resources, Methodology, Formal analysis, Conceptualization. **Masoud Yavarmanesh:** Writing – review & editing, Supervision, Resources, Project administration, Methodology, Data curation, Conceptualization. **Mohammad Reza Edalatian Dovom:** Supervision, Project administration.

## Declaration of competing interest

The authors declare that they have no known competing financial interests or personal relationships that could have appeared to influence the work reported in this paper.
